# Mechanochemical Functionalization of Polysaccharides via Catalytic Esterification for the Preparation of Superabsorbent Materials for Plant Growth Applications

**DOI:** 10.3390/ijms27188097

**Published:** 2026-09-11

**Authors:** Abdul Hafeez, Hao Wang, Romain Milotskyi, Gyanendra Sharma, Naoki Wada, Kenji Takahashi

**Affiliations:** 1 Division of Biological Science and Technology, Graduate School of Natural Science and Technology, Kanazawa University, Kakuma-Machi, Kanazawa 920-1192, Ishikawa, Japan; h-wang@se.kanazawa-u.ac.jp; 2 Faculty of Biological Science and Technology, Institute of Science and Engineering, Kanazawa University, Kakuma-Machi, Kanazawa 920-1192, Ishikawa, Japan; romain-mi@se.kanazawa-u.ac.jp (R.M.); sharmag-19@se.kanazawa-u.ac.jp (G.S.); naoki-wada@se.kanazawa-u.ac.jp (N.W.)

**Keywords:** mechanochemistry, polysaccharides, superabsorbent, okara, banana fibres, succinic anhydride, catalyst, bio-based

## Abstract

In this study we have developed a new approach to synthesize superabsorbent materials via derivatization of polysaccharides of soybean waste (okara) and banana fibres using mechanochemistry. These polysaccharides, in equal weight ratio, were derivatized with succinic anhydride (SA) in the thermo-mechanical reactive extrusion process in the presence of different amounts of catalysts K_2_CO_3_ and/or Zn(OAc)_2_. The reactions were performed at 130–140 °C for 10 min. Sequentially, after removal of unreacted SA, the derivatized polysaccharides were neutralized with NaOH, KOH and/or aq. NH_3_ to obtain superabsorbent materials. The esterified polysaccharides were crosslinked using glycidyl methacrylate (GMA) and/or *N*,*N*′-methylene *bis*acrylamide (MBA) and the effects were evaluated. The reactions were confirmed by attenuated total reflectance-Fourier transform Infra-red (ATR-FTIR) spectroscopic analysis and structural characterization was done by X-ray diffraction (XRD) and scanning electron microscopy (SEM). Among the catalysts, K_2_CO_3_ performed better than Zn(OAc)_2_, while among crosslinkers, MBA performed better than GMA crosslinker. The esterified polysaccharides showed optimum water absorption of 36.5 ± 2.2 g/g. Then the superabsorbent materials were used to grow maize plants, and the use of 0.4 wt.% functionalized SAPs in soil resulted in statistically significant increase in shoots length as compared to control, although no significant effect was observed on roots length.

## 1. Introduction

The use of renewable polysaccharides [1] to develop biodegradable superabsorbent polymers (SAPs) [2] has garnered attention as an alternative to conventional petroleum-based SAPs [3,4]. Nonedible polysaccharide from agricultural residues [5,6,7] and food industry byproducts are particularly attractive feedstocks due to their sustainability, abundance and rich composition of cellulose, hemicellulose, lignin, and proteins [8]. To successfully convert these raw polysaccharides into effective SAPs, it is necessary to introduce hydrophilic functional groups into their structures.

Among the various modification techniques, polysaccharides functionalization via succinoylation has proven advantageous. Homogeneous or heterogeneous esterification with succinic anhydride (SA) has a wide range of applications, such as in super water absorbents [9,10], ion exchangers [11], heavy-metal scavengers [12,13], oil absorbents [14], emulsifiers [15,16], cellulose nanofibers [17,18], reinforcing materials [19] for various polymer matrices, wound healing [20], drug delivery systems [21,22], and food packing materials [23]. Specifically for SAP development, the ring-opening esterification of polysaccharide -OH groups with SA yields half-ester with a pendant carboxyl group moieties [17,24] which can be converted to salts which mimic commercial SAPs for water absorption [25]. The high number of these carboxylate groups increases the hydrophilicity of the esterified polysaccharides, which drive swelling behaviour as well as impart superabsorbent properties [9].

Selecting appropriate raw materials and reaction methods is important for this succinoylation process. One of the candidates is banana pseudo-stem, an agricultural byproduct produced during the postharvest step that is rich in cellulose, hemicelluloses, lignin, and other non-structural polysaccharides [26,27]. Similarly, the other candidate, okara, is produced in large quantities as a byproduct of tofu and soymilk industry. It is not used as dietary material due to its anti-nutrient effect and low sensory perception, therefore posing a significant disposal problem [28]. It contains crude polysaccharide fibres composed of cellulose, hemicellulose, and lignin, along with 25% protein, 10–15% oil, and minimal starch or simple carbohydrates [28,29]. Thus, the utilization of banana pseudo-stem polysaccharides and okara to produce superabsorbent materials can not only increase profits for banana farmers and the industry, but also save costs relating to landfilling, ash treatment and environmental burden. To achieve succinoylation of polysaccharides, homogenous [30] and heterogenous [31,32] reaction systems have been developed including *N*-methyl-2-pyrrolidone/lithium chloride (NMP/LiCl) [10] or *N*,*N*-dimethylacetamide/lithium chloride (DMAc/LiCl) [33,34], imidazolium-based ionic liquids [30,35] such as 1-butyl-3-methylimidazolium chloride (BMIMCl) and 1-allyl-3-methylimidazolium chloride (AMIMCl), dimethyl sulfoxide/tetrabutylammonium acetate (DMSO/TBAA) [36] or dimethyl sulfoxide/tetrabutylammonium fluoride (DMSO/TBAF) [10], and dimethyl sulfoxide/1,8-diazabicyclo [5.4.0]undec-7-ene/CO_2_ (DMSO/DBU/CO_2_) [37], *N*,*N*-dimethylformamide (DMF) [38], pyridine [12], DMAc, DMSO [39], xylene [32] and toluene. However, utilizing a solvent free system during functionalization reaction would reduce the environmental burden. Furthermore, the catalysts used to derive this esterification present their own challenges. Basic catalysts like imidazole [40], pyridine and triethylamine (TEA) [33] are highly toxic, malodorous, and difficult to safely remove from the final product. Similarly, highly efficient catalysts such as 4-dimethylaminopyridine (DMAP) [35,41] and *N*-bromosuccinimide (NBS) [42] accelerate the reaction but are very expensive, severely limiting their economic viability for bulk commercial SAP production. Therefore, the development of a greener, cost-effective, and less toxic catalytic system remains a critical challenge in the sustainable production of polysaccharide-based SAPs. To address these limitations, inexpensive alkali metal salts [21,24,34,43] and metal-based Lewis acids [44,45,46] have emerged as promising, eco-friendly alternatives. Specifically, potassium carbonate [24] is a mild, low-toxicity solid base that has proven to be a highly effective catalyst for the esterification of carbohydrates, facilitating the deprotonation of hydroxyl groups. Furthermore, transition metal salts such as zinc acetate act as efficient esterification and depolymerization catalysts that can synergistically interact within biopolymer matrices during the modification processes [47].

Driven by the need for sustainable development, the aim of this work was to find more benign, and simpler approach for functionalization of polysaccharides. Therefore, we developed a green heterogeneous [48] polysaccharide succinoylation reaction. This study presents a strategy for the succinoylation of polysaccharides using a mechanochemical reactive extrusion system with the use of catalysts for successful esterification. Ultimately, this approach offers a viable pathway for the industrial-scale production of bio-based SAPs. To validate their practical utility, the prepared SAPs were tested in a preliminary and exploratory experiment for growth of maize plants in laboratory-controlled environment.

## 2. Results and Discussion

Biorefinery concepts targeting biomasses employ elaborate pretreatment steps for expensive fractionation of cellulose, hemicellulose and lignin. Using unfractionated biomasses as starting feedstock in simplified operations can considerably reduce processing efforts and increase the economic value of biomass residues [7]. Therefore, unfractionated raw materials of banana pseudo-stem fibres (BF) and okara were used for mechanochemical esterification reaction.

### 2.1. Mechanochemical Functionalization of BF and Okara

To align with green chemistry principles and minimize waste, raw okara and BF were utilized directly without prior protein extraction or chemical pretreatment, thereby preventing the generation of alkaline wastewater. While okara contains approximately 25% protein alongside 50% dietary fibres [49], these native proteins do not hinder the modification process. Instead, their abundant amino and hydroxyl functional groups actively participate in the esterification reaction, integrating covalently into the final SAP. Similarly, BF contains approximately 60–70% polysaccharides and about 12% lignin, alongside ash and extractives [50]. Utilizing these materials in their native state provides a rich, multi-component reactive mass for the solventless mechanochemical functionalization.

**Scheme 1:**
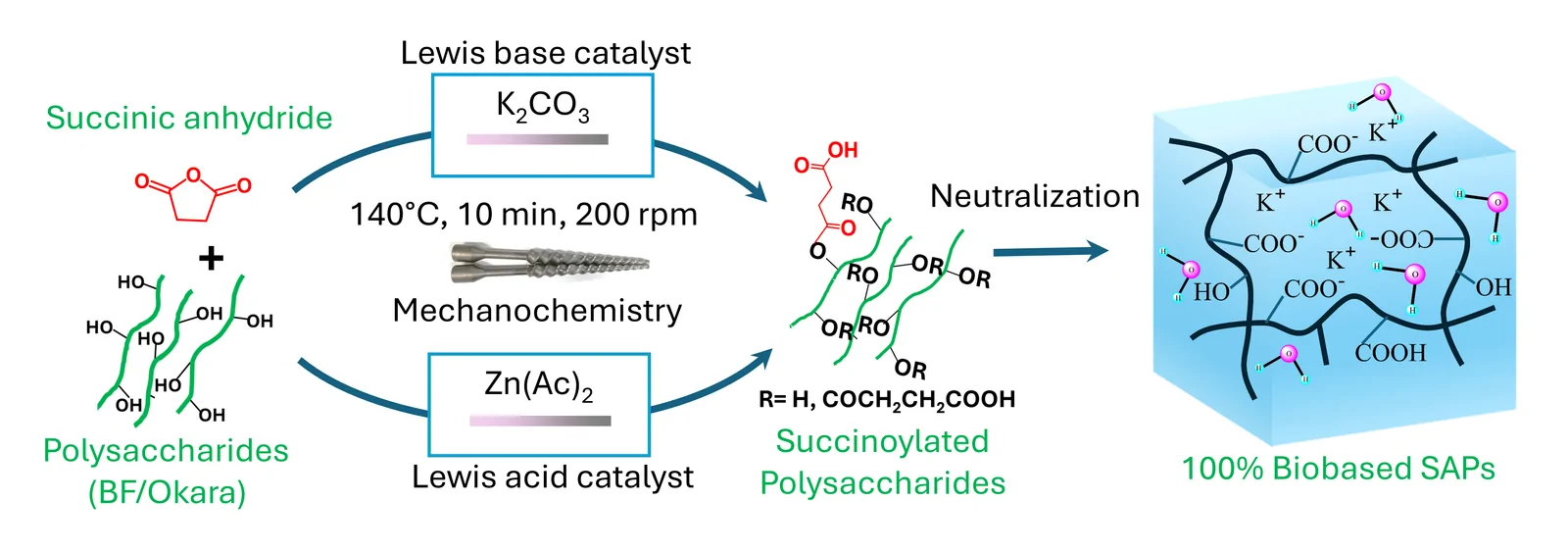
Illustration of mechanochemical succinoylation of BF/okara polysaccharides in the presence of K_2_CO_3_ and Zn(OAc)_2_ and conversion into superabsorbent materials.

All the reactions were performed at 140 °C for 10 min using microcompounder (15 mL) (MC 15, Xplore Instruments BV, Sittard, The Netherlands) except the SAP (Entry 9; Table 1) which was prepared using continuous extrusion process with a twin-screw extruder. Attenuated total reflectance-Fourier transform Infra-red (ATR-FTIR) spectroscopy was used to analyse the chemical structure of BF and okara polysaccharides before and after the mechanochemical esterification reaction. Esterification of BF/okara with SA opens up the cyclic anhydride ring and gives terminal carboxyl functional groups attached to -OH groups of polysaccharides as shown in Scheme 1.

**Figure 1:**
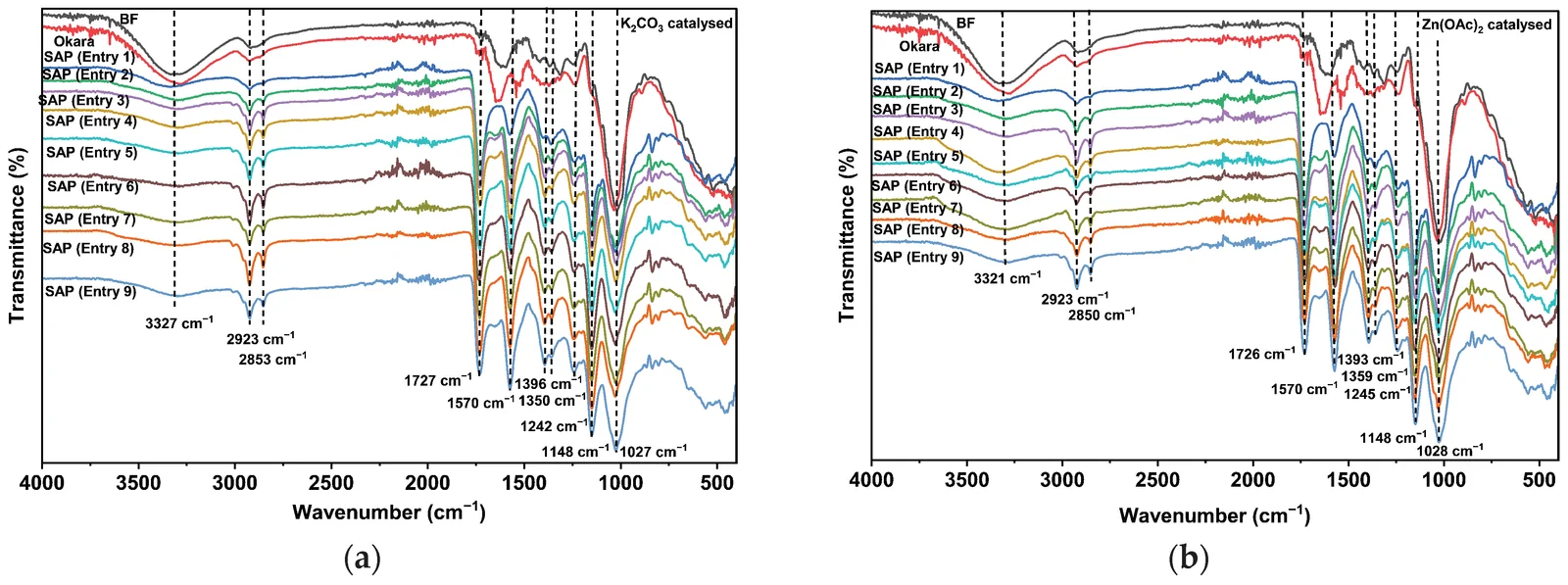
ATR mode FTIR spectra of raw materials and mechanochemically synthesized SAPs. Vertical dashed lines highlight major characteristics peaks to trace the functional groups transformation and successful mechanochemical esterification (**a**) BF, okara and K_2_CO_3_-catalysed SAPs (Entries 1–9; detailed formulations are provided in Table 1). (**b**) BF, okara and Zn(OAc)_2_-catalysed SAPs (Entries 1–9; detailed formulations are provided in Table 1).

The unmodified BF showed absorptions bands at 3330, 2900, 1634, 1438, 1315, 1160, 1105, 1052 and 1030 cm^−1^ which were characteristics of polysaccharide biomass. The band at 3330 cm^−^^1^ was attributed to O-H stretching and at 2900 cm^−1^ to CH stretching vibrations of polysaccharides. The bands at 1438 and 1315 cm^−1^ were associated with symmetric bending of CH methylene groups and to CH bending, respectively. The characteristics absorptions at 1160, 1105, 1052 and 1030 cm^−1^ originated from the glucopyranose rings of cellulose, specifically the C-O-C asymmetric stretching of glycosidic bonds, glucose ring stretching, and C-O stretching, respectively [51]. The carboxyl functions of the esterified polysaccharides were identified by ATR-FTIR. Figure 1 shows the ATR mode FTIR spectra of raw materials, BF and okara, along with all the SAPs prepared from these materials under different conditions and amounts of catalysts K_2_CO_3_ and Zn(OAc)_2_. The BF after mechanochemical esterification in the form of SAP (Entry 1) showed characteristics ester linkages with a carbonyl stretching peak at 1727 cm^−1^, simultaneously giving asymmetric stretching vibration of carboxylate anion (-COO^−^) at 1570 cm^−1^. Meanwhile, the polysaccharide skeleton preservation is evidenced by glycosidic asymmetric C-O-C stretching at 1148 cm^−1^, and skeletal C-O, C-C pyranose ring modes at 1027 cm^−1^. Similarly, the FTIR spectra of okara after mechanochemical esterification and neutralization in the form of SAP (Entry 2) showed the characteristics peaks as shown by SAP (Entry 1) prepared from BF confirming the successful esterification of okara polysaccharides. It is interesting to note that the SAPs (Entries 3–9) which were prepared with equal weight ratios of BF/okara also showed similar FTIR peaks.

The ATR-FTIR spectra of succinoylated samples before neutralization (Figure S1) showed the strong and broad carbonyl band at 1714 cm^−1^ with reduction in O-H band intensity at 3335 cm^−1^ [52]. After neutralization and H^+^ exchange of extruded materials with metal cations or NH_4_^+^ cations, the band at 1714 cm^−1^ split into two peaks, separating the ester -COO- carbonyl peaks at ~1727 cm^−1^ and carboxylate (-COO^−^) groups (conjugate form) peaks at 1570–1600 cm^−1^ [17]. The conversion of these pendant carboxylic groups into carboxylate form with NaOH, KOH or aq. NH_3_ transform these polysaccharides into SAPs. In principle, the larger the number of these carboxylate groups, the better the water absorption of the SAPs. The number of carboxylate groups beyond a certain limit make these polysaccharides hydrophilic enough and thus water soluble. The ATR-FTIR spectra of esterified BF (SAP Entry 1) indicated that compared to the original BF (Figure 1), the esterified BF presented a new strong band at ~1725 cm^−1^ and 1570 cm^−1^, corresponding to ester (-COO-) and carboxylate (-COO^−^) species, which provide clear evidence of succinoylation. Generally, ester (-COO-) and carboxylic acid (-COOH) bands appear at 1740 cm^−1^ and 1700 cm^−1^, respectively. However, in non-neutralized samples, overlapping of the two bands resulted in single peak centred at 1714 cm^−1^ (Figure S1). After neutralization the two bands arising due to -COO- and -COO^−^ groups clearly separated from each other (Figure 1) [53]. In Figure 1a, in K_2_CO_3_-catalysed SAPs, successful esterification is clearly marked by the decrease in broad O-H stretching vibration at 3330 cm^−1^, which indicates that native polysaccharide O-H groups were consumed in the reaction. Simultaneously, the C-H stretching vibrations at 2900 cm^−1^ and 2850 cm^−1^ intensify, which confirm the incorporation of aliphatic succinate backbone. Notably, the absence of a strong carboxylate (-COO^−^) band between 1560 and 1590 cm^−1^ (Figure S1) confirms that K_2_CO_3_ acts primarily as a catalyst rather than neutralizing the acid. Similarly, Figure 1b shows that the ATR-FTIR spectra of the Zn(OAc)_2_-catalysed reactions exhibited fundamental structural features of esterified with succinic anhydride as observed for both the non-catalysed and K_2_CO_3_-catalysed formulations.

### 2.2. Effect of Catalysts

To evaluate the influence of catalysts on the mechanochemical esterification reactions of BF/okara with SA, two different catalysts K_2_CO_3_ and Zn(OAc)_2_ were employed under similar reaction conditions. The resulting materials were systematically characterized to understand the impact of catalysts on chemical structure, COOH content, crystallinity, and swelling behaviour. K_2_CO_3_ is a mild Lewis base which has been reported in many studies for its catalytic effect in modification of polysaccharides [24,47], but it has not been systematically studied for catalytic effects in reactive extrusion process. It works by deprotonation of -OH groups on polysaccharides to enhance their nucleophilicity, which in turn facilitates the reaction by attacking on carbonyl groups of SA cyclic rings. On the other hand, zinc acetate is a low-hazard mild Lewis acid catalyst, which is widely used in organic catalysis for acylation/esterification reactions [45,47], implying that it can coordinate to carbonyls or hydroxyl groups for esterification. In comparison, the use of ZnCl_2_ in the esterification of cellulose is reported [54], but the use of ZnCl_2_ in the case of a reactive extruder would possibly generate highly corrosive HCl during esterification reaction that can damage the reactive extruder. That is why Zn(OAc)_2_ was chosen as a mild alternative to the ZnCl_2_ catalyst.

**Figure 2:**
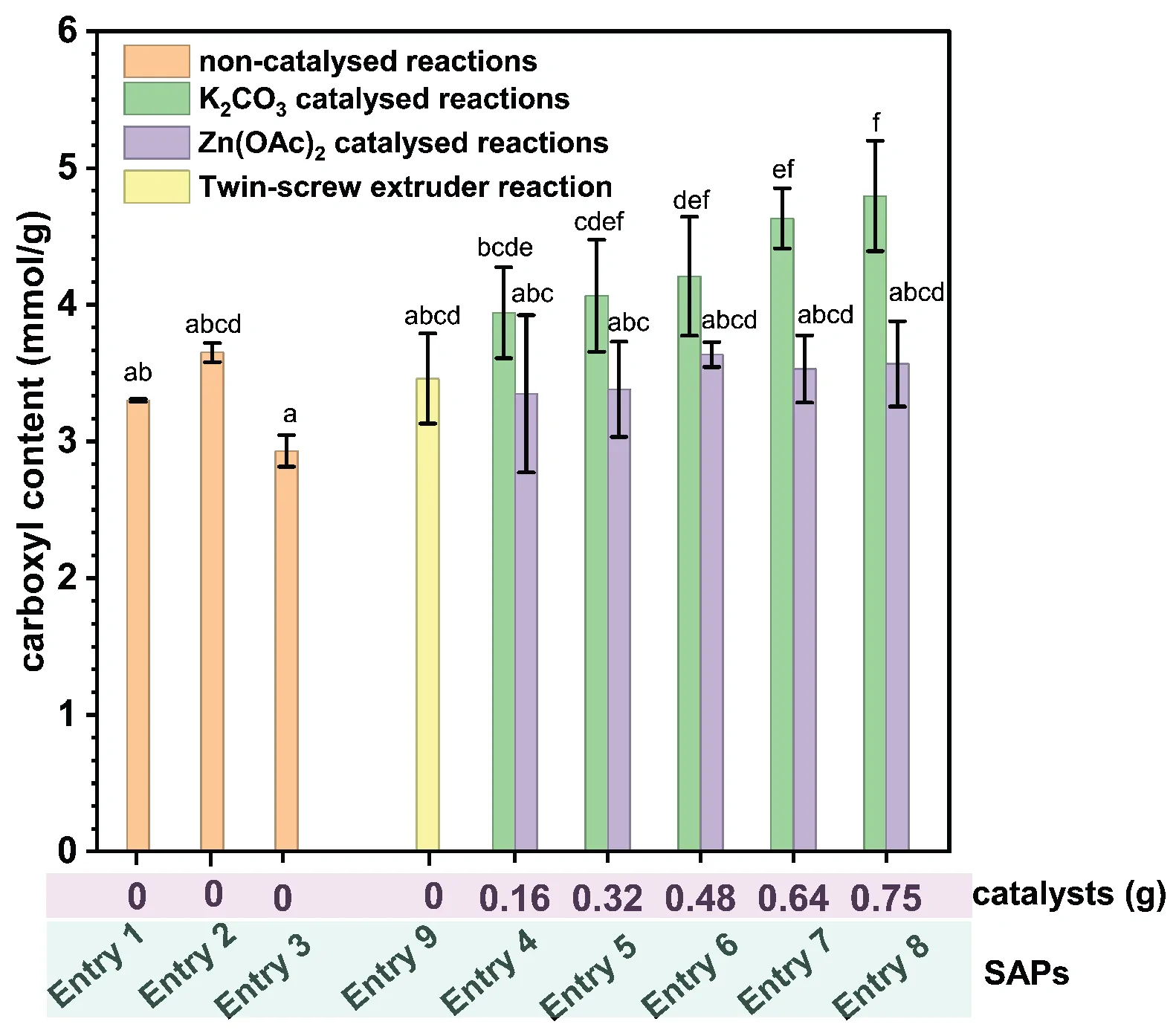
Comparison of carboxyl content (mmol/g) across varying reaction conditions. The data represents non-catalysed reactions (Entries 1–3) with variable composition of raw materials as presented in Table 1, reactions using varying mass loadings (0.16–0.75 g) of either K_2_CO_3_ or Zn(OAc)_2_ catalysts (Entries 4–8), and a non-catalysed reaction performed using a twin-screw extruder (Entry 9). The data are presented as mean ± standard deviation (*n* = 4). Error bars indicate standard deviation, and different lowercase letters means not sharing a common letter are significantly different (two-way ANOVA, *p* < 0.05, followed by Tukey’s HSD test).

It was noticed that with the increase in use of K_2_CO_3_ as a catalyst the peaks of ATR-FTIR spectra corresponding O-H groups at 3330 cm^−1^ decreased along with concomitant increase in 2900 cm^−1^ C-H stretching vibration. It was also observed that with an increase in amount of K_2_CO_3_ as catalyst from zero to 0.75 g, the COOH content, as measured by titration method, of the SAPs increased from 2.93 ± 0.11 mmol/g to 4.80 ± 0.40 mmol/g (Figure 2; Table S1). It suggested that K_2_CO_3_ acted as effective catalyst in increasing the degree of esterification. It was also noted that the amount of K_2_CO_3_ used was purely of catalytic amount, if it were able to neutralize the esterified materials, then two separate absorption bands would have appeared in the ATR-FTIR spectra of the SAPs due to -COO- and -COO^-^ functionalities. On the other hand, the use of the Zn(OAc)_2_ catalyst does not appreciably affect the ATR-FTIR spectra of esterified SAPs nor their COOH content. When the amount of Zn(OAc)_2_ catalyst was increased from zero to 0.75 g, only the modest increase in COOH content from 2.93 ± 0.11 mmol/g to 3.64 ± 0.09 mmol/g was observed. It suggested that although Zn(OAc)_2_ has been reported to be an effective catalyst in esterification reactions [47], in the case of free COOH groups its catalytic efficiency is reduced, probably due to formation of coordinate covalent bonds between COOH groups and Zn^2+^ cations. However, under present solventless functionalization conditions, it exhibited limited catalytic activity towards esterification with SA, as evidenced by -COOH content (mmol/g) (Figure 2). This might be attributed to its relatively low acidity and dissociation in solventless functionalization conditions and, therefore, to its limited ability to activate anhydride in the complex BF/okara polysaccharide matrix. In contrast, K_2_CO_3_ exhibited higher efficiency, resulting in higher -COOH content (mmol/g) (Figure 2). These results suggested that stronger or more reactive catalytic systems are required under heterogenous catalytic systems in solventless reactive extrusion processes during functionalization with SA for effective promotion of esterification of lignocellulosic materials. The mechanochemical functionalization of individual raw materials, BF and okara, yielded carboxylic content of 3.30 ± 0.01 mmol/g and 3.65 ± 0.07 mmol/g, respectively. This suggests that okara probably exhibits more reactivity towards SA than BF, which may be attributed to the higher inherent crystallinity of BF limiting reagent accessibility compared to the okara.

Although a direct comparison of COOH content is complicated by reaction conditions, the reaction system reported in the literature using molten SA for esterification of biomass for 6 h achieved relatively low carboxyl content [11]. In comparison with the literature, which revealed that the use of the K_2_CO_3_ catalyst for esterification with SA required extended reaction times [24], the present study enabled higher carboxyl content incorporation within a much shorter reaction time of 10 min, highlighting the efficiency of the catalytic mechanochemical reaction systems.

To compare the catalytic efficiencies of K_2_CO_3_ and Zn(OAc)_2_, a two-way ANOVA was conducted (Entries 1–9) on the carboxyl content. The analysis indicated a statistically significant main effect for the catalyst type (p<0.0001). The SAPs synthesized with K_2_CO_3_ yielded a higher overall mean carboxyl content (3.89 mmol/g) compared to those synthesized with Zn(OAc)_2_ (3.42 mmol/g). Furthermore, a significant interaction effect (p<0.0001) was observed, indicating that the disparity in efficiency between the two catalysts widened as the catalyst concentration increased (Entries 4–8). These statistical findings support the conclusion that K_2_CO_3_ is a more effective heterogeneous catalyst for the esterification of polysaccharides under solventless mechanochemical conditions. SAPs (Entries 7, and 8) demonstrate the superiority of K_2_CO_3_ catalyst; their specific letters (ef and f) (see Table S2 and Figure 2) do not overlap with the Zn(OAc)_2_ letters (abcd) for those same entries, indicating the difference at those specific concentrations.

### 2.3. Structural Changes

The structural changes in BF and okara polysaccharides after the mechanochemical esterification reaction were characterized by X-ray diffraction analysis. To isolate the chemical effects of the reactive extrusion from purely mechanical and thermal processing impacts, control trials were attempted by processing the pristine, dry okara and BF separately in the microcompounder without the catalysts and SA. However, a severe processing barrier was encountered due to the high thermal sensitivity of the unplasticized biomass. Raw okara is vulnerable to thermal degradation, with charring initiating at temperatures as low as 120 °C under dry conditions. During the control extrusion experiment, the high internal friction and intense mechanical shear within the confined 15 mL barrel generated severe viscous dissipation. This frictional heat caused an uncontrollable thermal overshoot, driving the internal melt temperature up to 165 °C (even after electrical heating elements were deactivated) and resulting in rapid material charring within 2 min. Consequently, obtaining a structurally intact ‘thermal-only’ control for comparative XRD analysis was technically unfeasible. Critically, this processing limitation highlights the dual role of the SA in this system; beyond acting as an esterification reagent, it functions effectively as a processing flux. It lowers internal torque and interfacial friction, thereby expanding the stable processing window and allowing the okara/BF polysaccharides to be successfully modified at 140 °C without undergoing thermal degradation.

**Figure 3:**
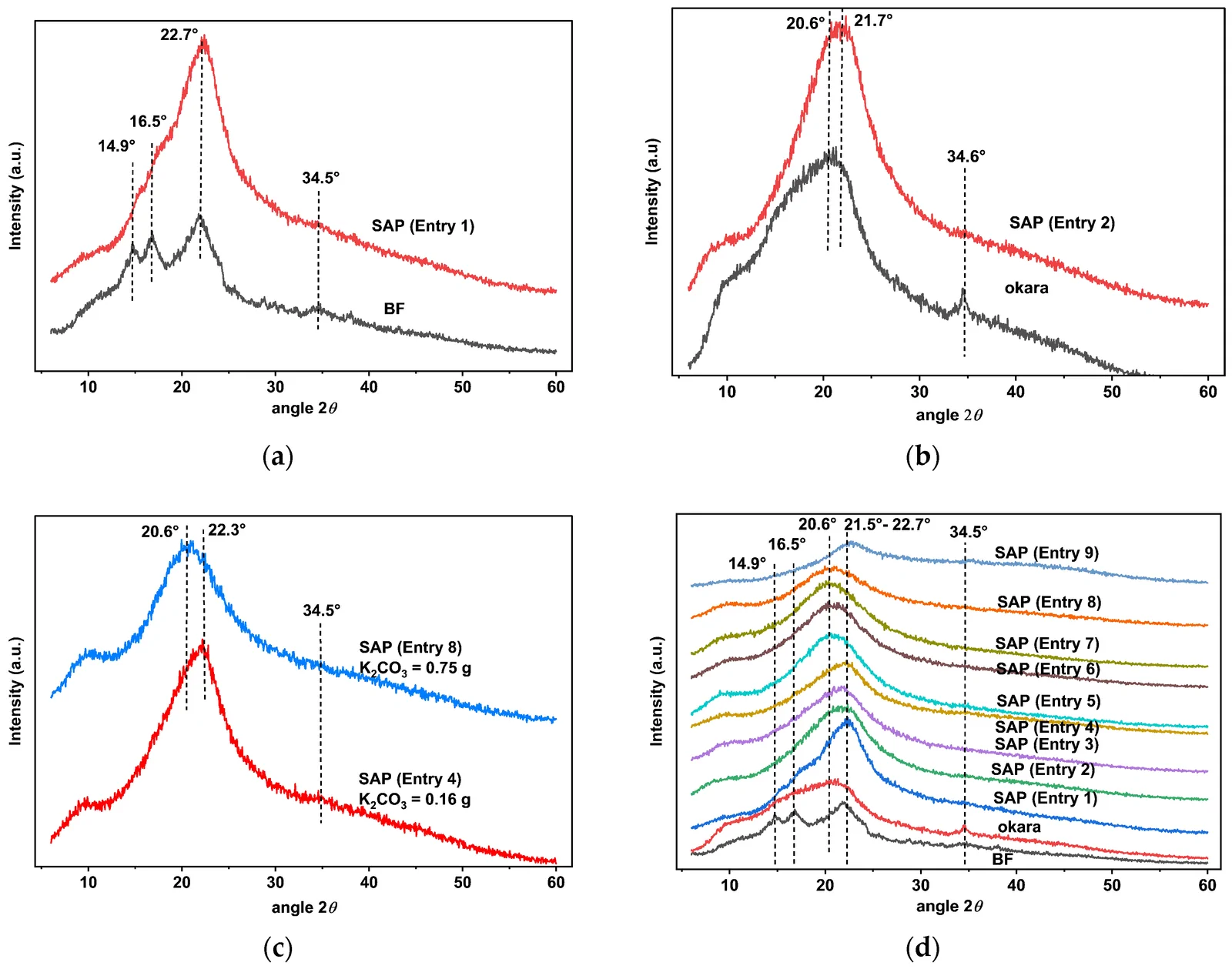
X-ray diffraction (XRD) patterns of raw materials and their mechanochemically synthesized SAPs after neutralization with aq. NH_3_. (**a**) Comparison of BF and its resulting SAP (Entry 1). (**b**) Comparison of raw okara and its resulting SAP (Entry 2). (**c**) Structural impact of catalyst concentration, comparing SAPs synthesized with low (Entry 4, 0.16 g K_2_CO_3_) versus high (Entry 8, 0.75 g K_2_CO_3_) catalyst amounts. (**d**) Comprehensive stacked diffractograms across the entire experimental matrix (Entries 1–9) and baseline raw materials. Vertical dashed lines with corresponding 2θ values indicate the positions of major characteristic diffraction peaks, illustrating the preservation, shift, or disruption of crystalline regions following the mechanochemical synthesis.

It was observed that the crystallinity of BF was largely disrupted by the mechanochemical reaction with SA (Figure 3a) even without the use of catalyst. This indicated that the mechanochemical reaction disrupted the native lignocellulosic structure of BF because rigid structures of lignin, hemicellulose and cellulose in native BF can absorb mechanical energy much more efficiently than pure form of cellulose [55]. Before the reaction BF showed distinct diffraction peaks at 14.9°, 16.5°, 22.7° and 34.5° which correspond to the 110, 1$\bar{1}$0, 200 and 004 planes, while after the reaction one broad peak was observed at 22.7° indicating maximum disruption of intramolecular crystalline regions [56]. The mechanical shear and compressive forces broke the rigid crosslinked bonding in BF and exposed the -OH bonds to the surface for functionalization. That is why it showed the carboxyl content value of 3.30 ± 0.01 mmol/g (Table S1). On the other hand, X-ray analysis of okara before and after succinylation reaction showed small differences in crystallinity when compared with native okara, which indicated that the okara and its SAP (Entry 2) are predominantly amorphous in nature (Figure 3b) [57]. It suggested that the crystalline structure of okara, which was composed of multiple non-crystalline components along with cellulose and hemicellulose, remain largely unchanged before and after mechanochemical modification. With increasing the amount of K_2_CO_3_ catalyst from 0.16 g to 0.75 g, the crystallinity peak at ~22.3° shifted slightly to shorter angle at ~20.6° as shown in Figure 3c. Furthermore, after neutralization of COOH groups to carboxylate groups, they further diminish the crystallinity of modified materials by making the fibrils expand due to negatively charged surface. All the SAP samples either neutralized with KOH, NaOH or aq. NH_3_ showed almost similar X-ray diffraction patterns as SAP (Entry 1). The XRD diffractograms of all the SAPs (Entry 1 to Entry 9) prepared using K_2_CO_3_ catalyst and neutralization with NH_3_ are shown in Figure 3d.

### 2.4. Morphological Changes

**Figure 4:**
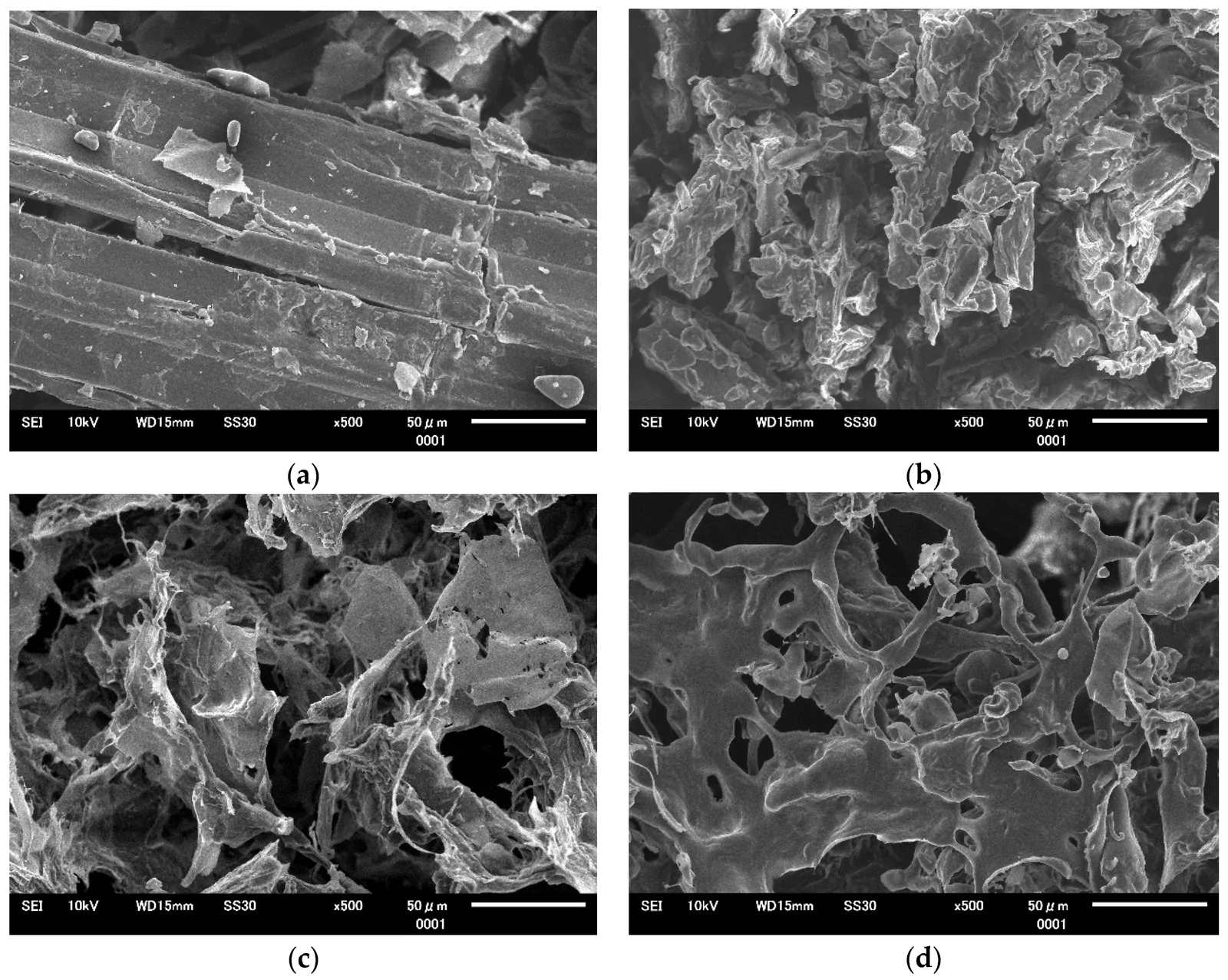
SEM micrographs of the raw materials and their corresponding SAPs: (**a**) BF, (**b**) okara, (**c**) SAP corresponding to Entry 6, and (**d**) SAP corresponding to Entry 8.

The SEM micrographs of BF, okara, and SAPs (Entry 6 and Entry 8; Table 1) are shown in Figure 4. The micrograph in Figure 4a shows that the BF has vascular bundles, cellulosic fibrils and cell wall structures clearly visible before the mechanochemical modification with SA. After the reaction, the structure turned into microfibrils, and cell wall structures collapsed yielding disoriented interconnected networks. Figure 4b shows the micrograph of okara which had a compact type protein matrix structure in which microfibrils are embedded [57]. It was observed that with increasing amounts of the K_2_CO_3_ catalyst, the microporous structures of SAPs become more pronounced. This effect can be seen in the SEM micrograph (Figure 4c) of the SAP (Entry 6) prepared using a 0.48 g K_2_CO_3_ catalyst. After mechanochemical esterification BF/okara turned into macroporous architectures with inner interwoven structures which provided the large surface area, resulting in a better water–SAP interface and higher water absorption. Further increases in the catalyst resulted in the larger interconnected macroporous structures breaking up into layers of microfibrils (Figure 4d).

### 2.5. Measurement of Free Water Uptake

It is important to note that after mechanochemical succinoylation, the materials have pendant -COOH groups which show poor hydrophilic properties. Therefore, free water uptake capacity of synthesized SAPs was investigated using the tea bag method [57,58] after neutralization of acidic carboxyl groups with different bases, e.g., 4 wt.% KOH, 4 wt.% NaOH or dilute aq. NH_3_ solutions.

It was found that the water absorption of succinoylated polysaccharides was different when neutralized with different bases. The proton exchange of COOH groups with cations made the -COO^−^ groups hydrophilic. There are multiple factors which govern the water absorption behaviour of SAPs such as pH, ionic strength of surrounding solutions, and the dissociation of carboxylate functionalities. Since the swelling capacity of these acidic functionalized polysaccharides is pH dependent. Under low-pH conditions, the carboxylic groups remain mostly protonated (-COOH), forming hydrogen bonds that restrict water uptake. However, in a sufficiently high-pH environment exceeding the Pk_a_ of the -COOH groups, these moieties undergo in situ ionization into carboxylate anions (-COO^−^). The resulting electrostatic interactions and increased osmotic pressure expand the polymer network, allowing the swelling capacity to be comparable to the neutralized SAPs [59]. The enhanced water uptake of the neutralized SAP is governed by the physical chemistry of polyelectrolyte networks. Rather than a simple dissolution of completely undissociated salt moieties, the functionalities of SAPs exist in an equilibrium between associated ion pairs and separated counterions. As water penetrates the network, the dielectric constant of the internal polymer phase rises, shifting this equilibrium toward a higher degree of ion separation. The fixed charges along the polysaccharides backbone and separated mobile ions create unequal distribution of charges in the SAPs network, creating a Donnan osmotic pressure that increases the water uptake to balance mobile ions difference between the SAPs and external solutions. The increased swelling capacity is primarily attributed to the high concentration of fixed carboxylate anions within the network. While direct electrostatic repulsion between these fixed charges is largely screened by mobile counterions (Debye length < 1 nm), the presence of these counterions creates a significant mobile ion concentration gradient between the hydrogel matrix and the external medium. This induces a strong Donnan osmotic pressure, which acts as the principal thermodynamic driving force for network expansion and water uptake. The ionic strength of surrounding solutions strongly influences the swelling through additional contributions. Higher ionic strength of external solutions compresses Donnan layer, reducing the osmotic pressure difference, and thus swelling. That is why SAPs have reduced swelling capacity in salt solutions. Since the soil also contains different salts and solutes, the swelling capacity of the SAPs in soil is also expected to decrease [60,61].

**Figure 5:**
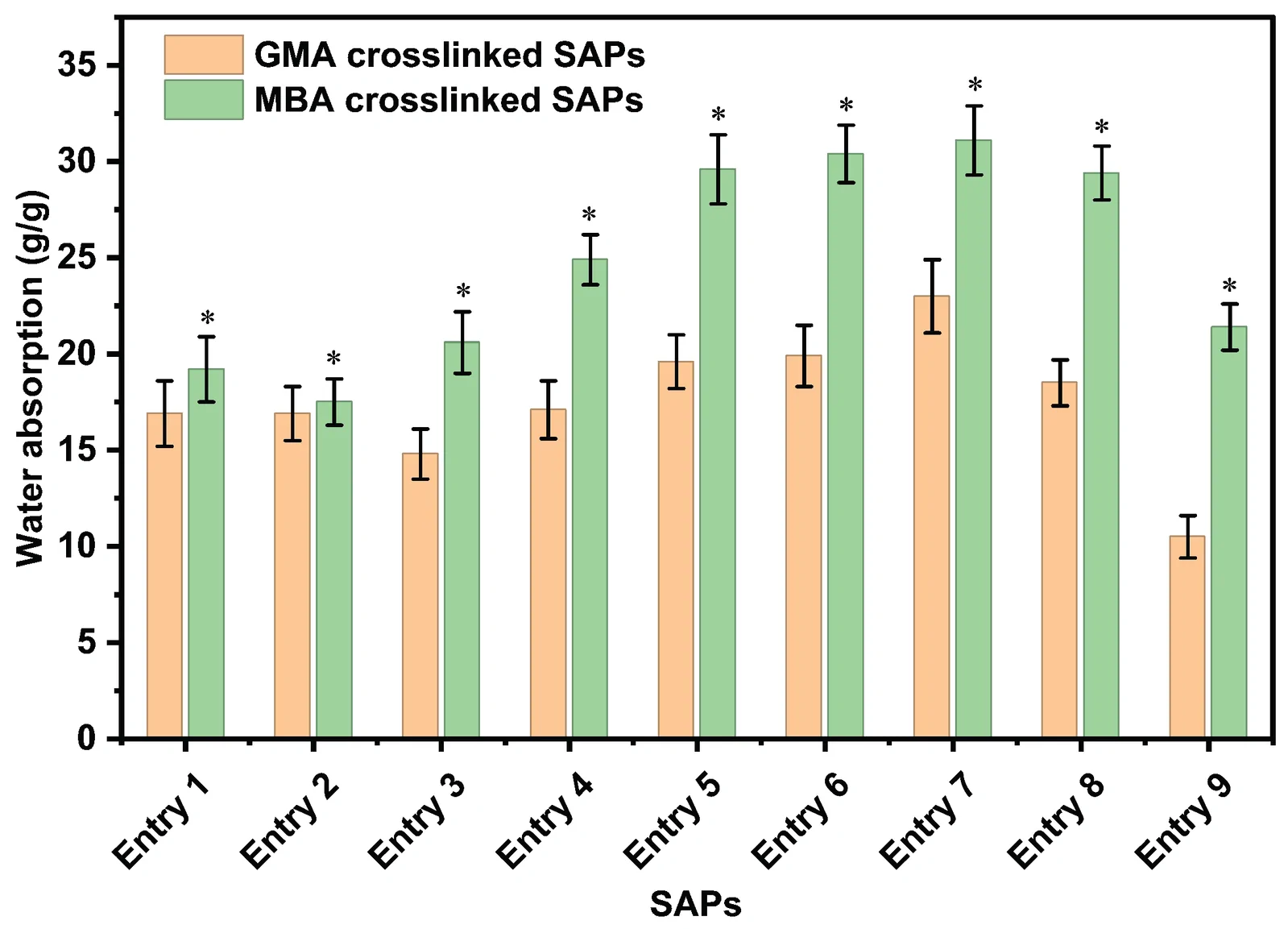
Water absorption of SAPs prepared using K_2_CO_3_ catalyst and crosslinked with GMA and MBA across different formulation conditions (Entries 1–9; detailed raw material compositions and K_2_CO_3_ catalyst amounts are listed in Table 1). Data are expressed as mean ± standard deviation (n=3). Asterisks (*) indicate statistically significant difference between the two crosslinking agents (p<0.05, two-way ANOVA followed by Tukey’s HSD post hoc test), showed that MBA crosslinked SAPs demonstrated a higher overall water absorption capacity than GMA crosslinked SAPs across all the Entries 1–9.

Overall, among all the SAPs except Entry 1 (Table 2), the SAPs neutralized with 4 wt.% KOH showed the highest water absorption. The SAP (Entry 7) synthesized using 0.64 g K_2_CO_3_ catalyst exhibited maximum water absorption of 36.5 ± 2.2 g/g in all the samples neutralized with aq. KOH (Table 2). Similarly, the samples prepared using Zn(OAc)_2_ as a catalyst showed relatively better water absorption when neutralized with aq. KOH as compared to aq. NaOH, albeit not significantly different. The SAPs synthesized using K_2_CO_3_ catalysts were attempted to crosslink with GMA and MBA as detailed in the Materials and Methods section and neutralized with aq. NaOH. Studies show that the degree of crosslinking controls the elastic resistance to swelling. The higher crosslink density restrains network expansion and reduces equilibrium swelling, whereas lower crosslinking increases swelling capacity [62]. It was observed that the water absorption of the SAPs prepared with GMA and MBA decreased compared to the SAPs synthesized without these agents. A comparison revealed that the addition of GMA resulted in a relative reduction in water absorption than MBA (Figure 5; Table S3). While a reduction in water uptake is indirect evidence and does not definitively demonstrate the formation of covalent crosslinks, it is consistent with the proposed crosslinking network formation. Assuming this network formation occurs, the relative reduction observed with GMA is hypothesized to stem from its potentially higher reactivity in this system compared to MBA. Another factor that might influence this proposed reaction is the physical state of the agents; as a liquid, GMA may distribute more uniformly within the mixture than the solid MBA, which could theoretically lead to a more homogenous network structure. The subtle differences in water absorption of different SAPs may be attributed to the heterogeneity of mechanochemical reactions which possibly make some parts of materials “hot spot” of COOH functionalities while others have sparce functional groups. The samples neutralized with dilute aq. NH_3_ leached out of the tea bag and the remaining insoluble fraction exhibited relatively less water absorption. Still, the SAP (Entry 7) showed the modest water absorption of 35.8 ± 2.3 g/g.

A two-way ANOVA was conducted to assess the effects of crosslinker type (GMA vs. MBA) and formulation variations (Entries 1–9) on the water absorption capacity of the SAPs. The analysis revealed a statistically significant main effect for the crosslinker type (p<0.0001), with MBA crosslinked SAPs exhibiting a higher overall water absorption compared to GMA crosslinked SAPs. Furthermore, a significant interaction effect (p<0.0001) indicated that the magnitude of MBA’s performance varied depending on the specific synthesis parameters of each entry. These results confirm that MBA serves as a better crosslinking agent than GMA for polysaccharide-based SAP.

While commercial, purely synthetic petrochemical-based SAPs routinely have swelling capacities exceeding 300 g/g, their production carries environmental liabilities and poor biodegradability [3,63]. In contrast, for fully bio-based, unfractionated polysaccharides such as the okara and BF hydrophilic materials developed here with an equilibrium water uptake of ~36.5 g/g marks a significant enhancement over the water absorption of unmodified raw biomass (typically <10 g/g). As contextualized in Table 3, this capacity is comparable to established polysaccharide-based SAPs and composites. Therefore, in alignment with the broader applied literature for sustainable agricultural and soil-conditioning matrices, the term ‘superabsorbent polymer’ is fitting, reflecting the material’s capacity to absorb water many times as compared to its dry mass.

### 2.6. Growth of Maize

**Figure 6:**
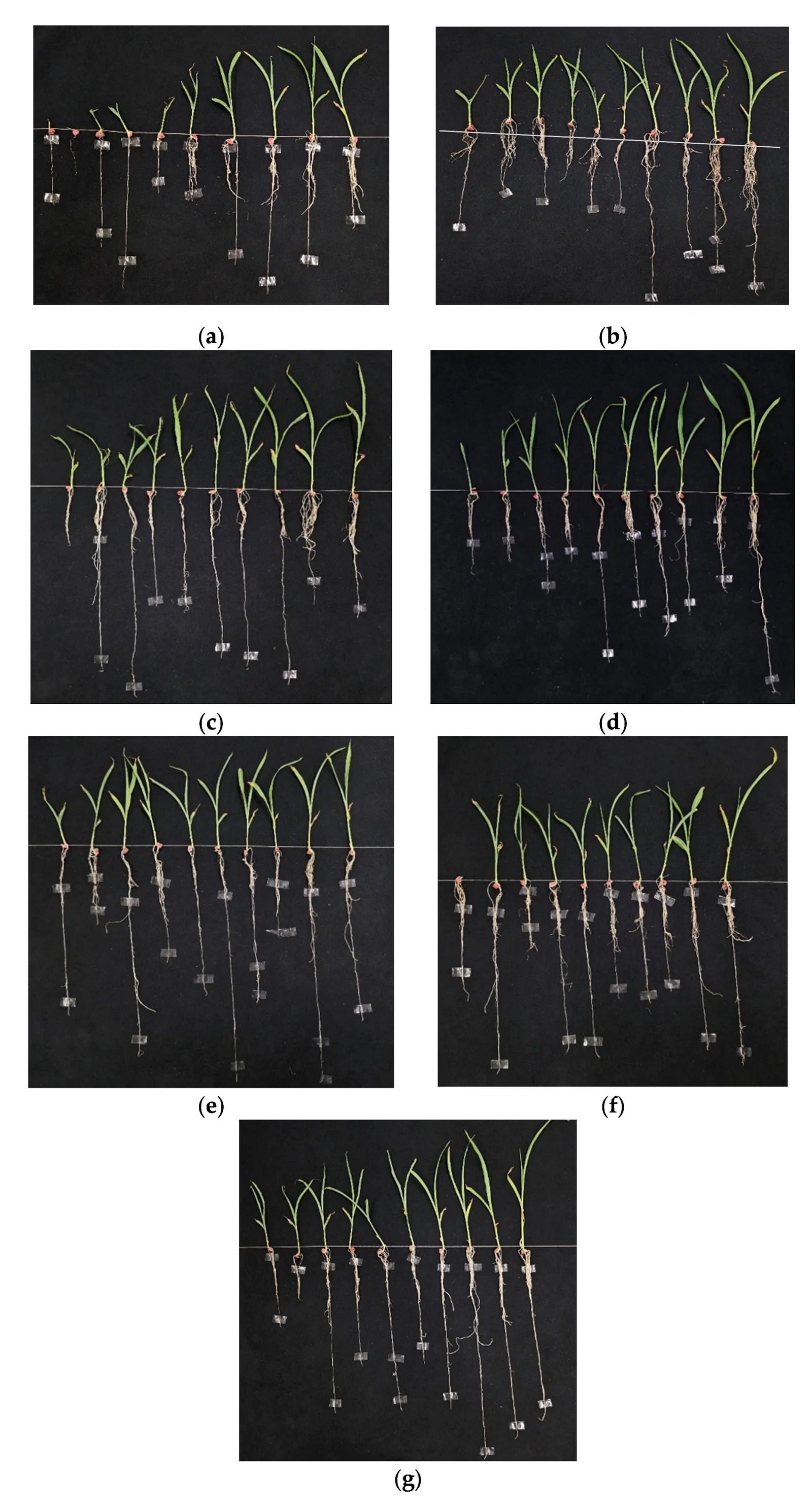
Growth of maize plants in vegetable garden soil. (**a**) Control (maize plants grown in soil without SAP addition), (**b**) maize plants grown in 0.1 wt.% untreated SAP, (**c**) maize plants grown in 0.2 wt.% untreated SAP, (**d**) maize plants grown in 0.4 wt.% untreated SAP, (**e**) maize plants grown in 0.1 wt.% treated SAP, (**f**) maize plants grown in 0.2 wt.% treated SAP, (**g**) maize plants grown in 0.4 wt.% treated SAP.

**Figure 7:**
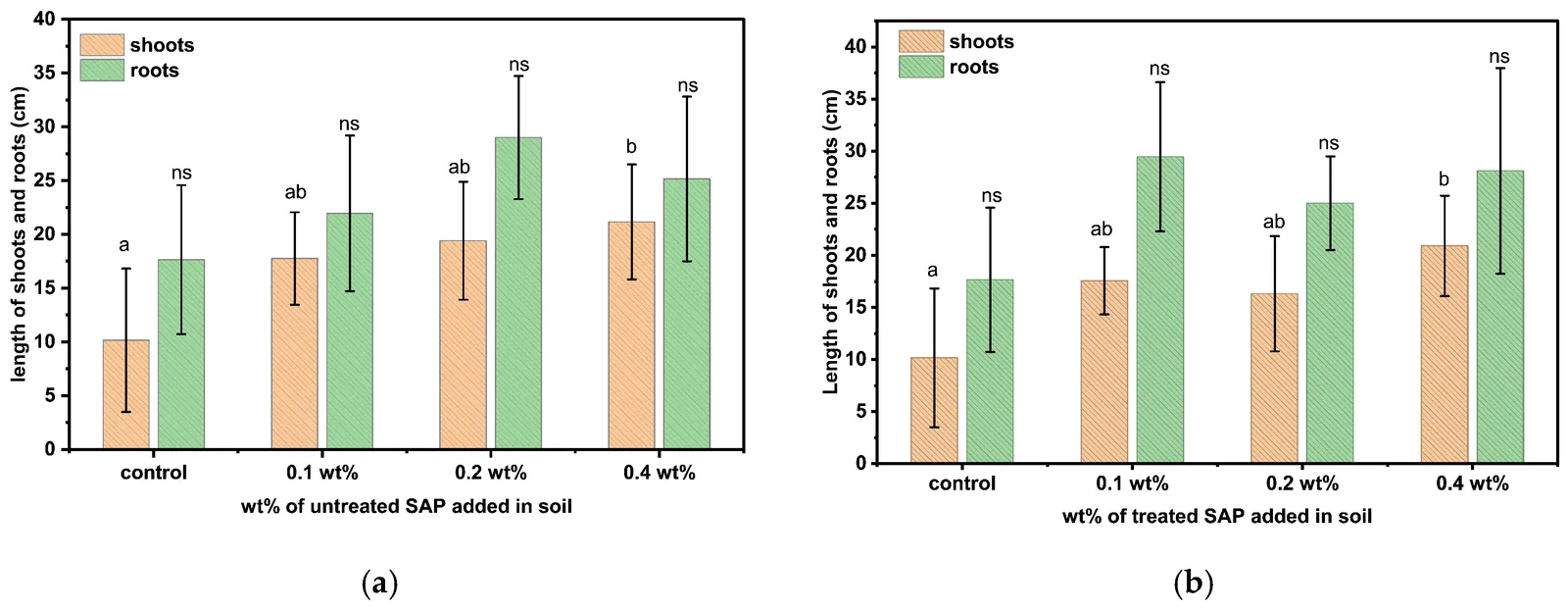
Effect of untreated SAP and treated SAP on length of maize shoots and roots (cm), (**a**) growth in soil with untreated SAP, (**b**) growth in soil with treated SAP. Data are presented as mean ± standard deviation (*n* = 5). Different lowercase letters above the shoot bars indicate significant differences among treatments according to one-way ANOVA followed by Tukey’s HSD test (*p* < 0.05). Statistically no significant differences were observed among root lengths (*p* > 0.05) and are labelled as ‘ns’.

The incorporation of SAPs into the vegetable garden soil positively affected the growth of maize plants in plantation experiment compared to maize plants grown in soil without SAP supplementation (control). Maize plants were grown in 0.1 wt.%, 0.2 wt.% and 0.4 wt.% SAP-added soil with both “treated” and “untreated” SAPs. Untreated SAP refers to the SAP obtained from twin-screw extruder after reaction and used without purification and neutralization, while treated SAP refers to the SAP processed for purification and neutralization. The addition of both untreated and treated SAPs generally enhanced maize growth compared with the control (Figure 6 and Figure 7). For untreated SAP, both shoots and roots length increased with increasing SAP concentration, reaching the highest mean values at 0.4 wt.% SAP. One-way analysis of variance (ANOVA) revealed a significant effect of SAP concentration on shoot length (one-way ANOVA, *F*_(3,16)_ = 3.86, *p* = 0.0297). Tukey’s honestly significant difference (HSD) test further showed that the 0.4 wt.% treatment produced greater shoot growth than the control, whereas the 0.1 wt.% and 0.2 wt.% treatments were not different from either the control or the 0.4 wt.% treatment. On the other hand, although roots length showed an increase with SAP application, the differences among treatments were not statistically significant (*p* > 0.05), indicating greater variability in roots growth during the experimental period (Figure 7a). A similar maize plant growth effect was seen for the treated SAP. Shoot length increased with increasing SAP concentration, with the greatest mean value obtained at 0.4 wt.%. Statistical analysis (one-way ANOVA, *F*_(3,16)_ = 3.71, *p* = 0.0336) again demonstrated a significant effect of SAP concentration on shoot growth, while root length did not differ among treatments. These results suggest that the beneficial effect of SAP application was higher for shoot development than for root elongation during this preliminary and exploratory growth experiment. Since experiment was conducted without watering the plants, the better shoot growth observed in maize plants grown in treated SAP-added soil may be due to the hydrophilic nature of the SAP.

Furthermore, it is important to note that the untreated SAP utilized in this study contained residual succinic anhydride from the functionalization process, which upon contact with water is converted into succinic acid. Since succinic acid is known to affect plant metabolism and may exert a growth-stimulating effect [69,70], the presence of unreacted succinic anhydride and succinic acid may represent a potential confounding factor in this experiment. Consequently, the observed plant responses cannot be attributed exclusively to the physical water-retention capabilities of the SAP, and these agricultural trials should be interpreted as preliminary and exploratory. Under these specific 10-day experimental conditions, the addition of SAP positively influenced plant height. Overall, these preliminary findings suggest that the SAP might have potential as a soil additive to support maize plant growth during short-term water withholding.

**Figure 8:**
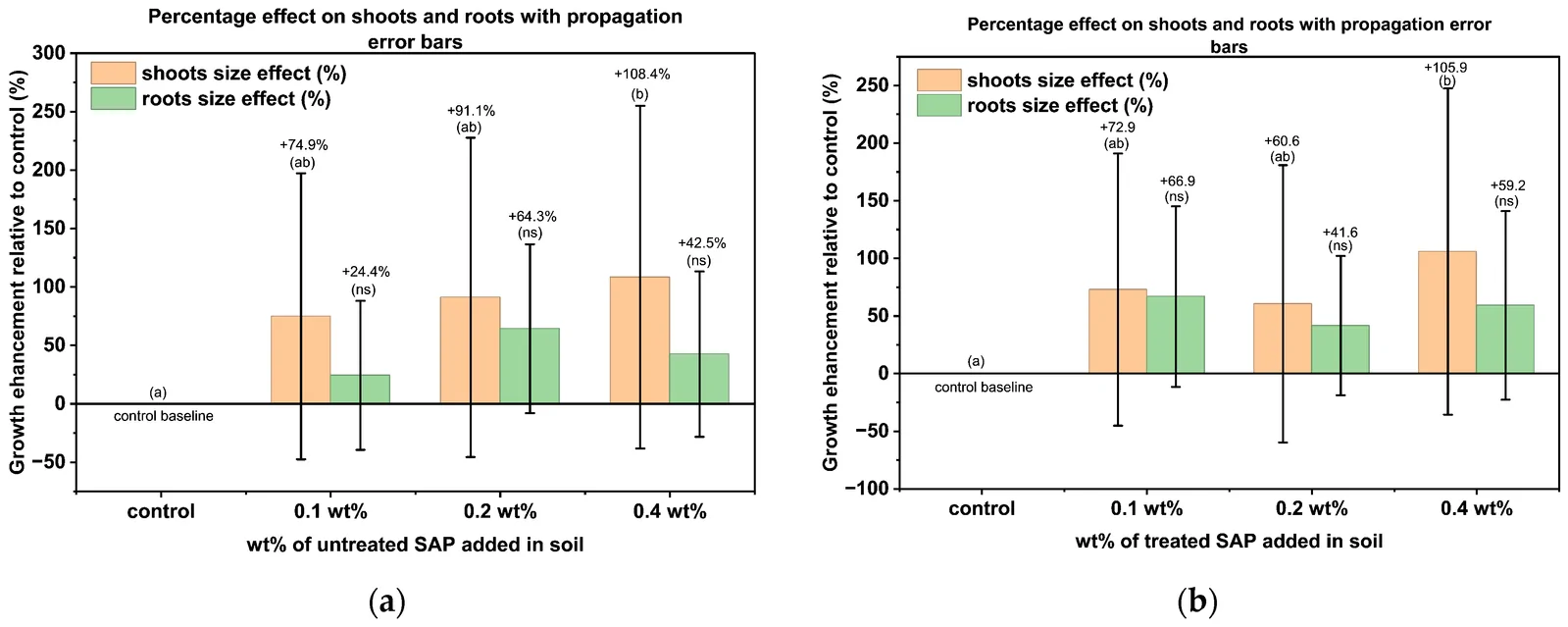
Percentage growth enhancement of plant shoots and roots relative to a control baseline, demonstrating the effects of varying concentrations (0.1 wt.%, 0.2 wt.%, and 0.4 wt.%) of superabsorbent polymer (SAP) in soil. (**a**) Growth enhancement using untreated SAP added to the soil. (**b**) Growth enhancement using treated SAP added to the soil. Error bars represent propagation errors. Value annotations above the bars indicate the specific percentage increase, accompanied by statistical significance markers (e.g., ‘a’, ‘b’, ‘ab’, or ‘ns’ for not significant).

The application of both untreated and treated SAP to soil consistently enhanced the growth of plant shoots and roots relative to the control. Across all tested concentrations (0.1, 0.2, and 0.4 wt.%), shoot growth experienced a higher percentage increase than root growth (Figure 8), with the maximum enhancement occurring at the 0.4 wt.% concentration for both untreated (+108.4%) and treated (+105.9%) SAPs. Although SAP application positively affected root size, the comparatively modest gains suggest the treatment primarily stimulates above-ground biomass development.

### 2.7. Comparative Techno-Environmental Considerations

To evaluate the real-world viability and sustainability of the developed SAP, its core properties must be contextualized against standard commercial polyacrylate-based SAPs. While commercial polyacrylate SAPs dominate in terms of sheer swelling capacity (routinely >300 g/g), their reliance on petrochemical feedstocks presents severe environmental liabilities, notably their persistence as microplastics and poor soil biodegradability [3,63]. In contrast, the okara- and BF-based materials developed in this study prioritize ecological safety and lifecycle sustainability (Table 4). By utilizing unfractionated agricultural waste, the synthesis circumvents both the toxic precursors of acrylics and the energy-intensive fractionation required for purified biopolymers. Economically, while commercial SAPs benefit from mature economies of scale, the raw materials for the developed SAP are essentially zero-cost waste streams. Ultimately, the ~36 g/g swelling capacity of the okara/BF SAPs, although lower than synthetic SAPs, marks a significant enhancement over raw biomass (<10 g/g) with minimal associated environmental tax.

**Table 4:** Comparative evaluation of synthesis, swelling, degradability, cost and environmental impact.

Matric	Developed SAP (BF/Okara)	Commercial Polyacrylate SAPs
Synthesis	Green synthesis utilizing unfractionated biomass (banana pseudo-stem/okara); avoids toxic crosslinkers	Petrochemical-derived; relies on radical polymerization involving toxic monomers (e.g., acrylic acid) and crosslinkers.
Swelling	~36 g/g (equilibrium water uptake)	>300 g/g (highly optimized for maximum capacity)
Degradability	Fully bio-based; susceptible to rapid microbial degradation and natural enzymatic breakdown in soil.	Highly recalcitrant; degrades at near-zero rates (<1% over several months) in agricultural soils [3,63].
Cost	Low-cost raw material due to waste valorization; synthesis bypasses complex fractionation steps.	Cheap at industrial scale due to mature manufacturing but carries high hidden environmental costs.
Environmental Impact	Net-positive; valorizes agricultural waste (Okara) and acts as a safe soil-conditioning matrix.	High liability; contributes to microplastic pollution and potential localized toxicity from unreacted monomers.

## 3. Materials and Methods

The banana pseudo-stem chips of approximate size of 5 cm were purchased from One Planet Cafe Ltd. (Tokyo, Japan). The chips were hot air oven dried at 70 °C before pulverizing into 0.3 mm fibre size powder, henceforth called banana fibres (BF) using swinging hammer crusher (Sansho Industry Co., Ltd., Osaka, Japan). Okara powder having ultra-fine particle size of 150 mesh was purchased from Yutec Co., Ltd. (Hokkaido, Japan). Succinic anhydride (SA) (purity > 97%), KOH (purity > 84%), NaOH (purity 97.0%), potassium peroxodisulfate (KPS, purity 98.0%) and Ethyl acetate (99.8%) were purchased from Kanto Chemical Co., Inc. (Tokyo, Japan). Potassium carbonate (purity > 99.0%), Zinc acetate (purity > 98.0%) and Glycidyl methacrylate (GMA, purity > 95.0%) were purchased from Tokyo Chemical Industry (TCI, Tokyo, Japan). *N*,*N*′-methylene *bis*(acrylamide) (MBA, ≥99%) was purchased from FUJIFILM Wako Pure Chemical Co., (Osaka, Japan). All the chemicals were used as received without further purification.

For lab-scale experiments benchtop kneader Xplore Series microcompounder MC 15 HT (Xplore Instruments BV, Sittard, The Netherlands) was used for mechanochemical functionalization of BF and okara polysaccharides with SA to prepare SAPs. For larger-scale mechanochemical synthesis of SAP a co-rotating twin-screw extruder (Omega 30H co-rotating twin-screw extruder, STEER JAPAN Co., Ltd., Tokyo, Japan) was used. The maize seeds, Gokkan Corn, were purchased from Atariya Farm Co., Ltd. (Chiba, Japan).

### 3.1. Mechanochemical Succinoylation of BF/Okara and Conversion into SAP

For the mechanochemical succinoylation reaction, pre-mix of BF, okara, SA and catalyst were prepared. The pre-mix were prepared by taking 3.2 g of BF and okara, which were taken in equal weight ratios with 10.3 g SA along with varying amount of catalyst (K_2_CO_3_ or zinc acetate) ranging from 0.16 g to 0.75 g (Table 1). Once all the reactants were thoroughly mixed using grinding mixer (Crush Millser, Iwatani Corporation, Osaka, Japan), they were gradually introduced into the pre-heated barrel of 15 mL microcompounder set to operate at 140 °C and 200 rpm. After kneading the reactants for 10 min, the crude product was collected, cooled and grinded again into powder. Afterwards, it was washed several times with ethyl acetate to remove unreacted SA. Subsequently, it was washed with MeOH to remove any traces of unreacted SA. Once purified the product was divided into two parts, one part was used for measuring -COOH functionalities and the other part was neutralized with either 4 wt. % KOH or NaOH solution or dilute aq. NH_3_ until the pH was neutral. Thus, neutralized SAP materials were filtered using filter paper and Büchner funnel under gentle suction and washed with distilled water to remove impurities. Afterwards, the SAPs were dried using vacuum drier for 78 h before measuring water absorption and other characterization.

For larger-scale mechanochemical synthesis of SAP (Entry 9) a co-rotating twin-screw extruder was used. The extruder had a screw diameter of 30 mm and a length-to-diameter (L/D) ratio of 60:1. The screw configuration consisted of conveying and kneading elements arranged to ensure effective mixing and shear. The temperature profile across the heated barrel zones of twin-screw extruder was maintained at 130–135 °C. Polysaccharides (BF and okara) premixed with SA, without the catalyst, were fed into the extruder through a gravimetric feeder. The processing was performed at a screw speed of 50 rpm with a feed rate of 3.6 kg/h. The residence time was approximately 10 min. The extruded materials were collected, cooled to room temperature, and further processed for characterization, measurement of water absorption and application for maize plant growth. The weight percent gain (WPG) of BF/okara after mechanochemical esterification with SA was calculated by Equation (1):

(1) $$WPG\;(\%)=\frac{W_{p}-W_{b}}{W_{b}}\times 100$$

where $W_{p}$ is the weight (g) of purified and dried product before neutralization step and $W_{b}$ is the weight (g) of BF/okara used for modification. WPG was utilized as a preliminary, macroscopic indicator for functionalization. The quantitative evaluation of esterification was derived from replicated titration for carboxyl content (*n* = 4) as described in Section 3.2.4.

#### 3.1.1. Crosslinking of Succinoylated BF and Ok

After purification without neutralization the 0.5 g of succinoylated product was crosslinked either using glycidyl methacrylate or *N*,*N*′-methylenebis(acrylamide). The procedure for both is described below.

#### 3.1.2. Crosslinking Using GMA

In 20 mL glass vial equipped with magnetic stirrer, 0.5 g succinoylated product was dispersed in 10 mL dehydrated DMSO at 60 °C for 1 h. Afterwards, 15 µL GMA crosslinker was added followed by the addition of 2.5 mg K_2_S_2_O_8_ dissolved in 1 mL DMSO. The mixture was stirred for 6 h at 70 °C, followed by the precipitation in 30 mL acetone. Filtered the crosslinked product, dispersed in water, neutralized and freeze dried. Then water absorption was measured along with other characterization.

#### 3.1.3. Crosslinking Using MBA

Similar to crosslinking reaction using GMA, in a 20 mL glass vial equipped with magnetic stirrer, 0.5 g succinoylated product was dispersed in 10 mL dehydrated DMSO at 60 °C for 1 h. Then, 2.5 mg K_2_S_2_O_8_ dissolved in DMSO was added to the vial while maintaining the temperature at 60 °C. Subsequently, 15 mg MBA dissolved in another 1 mL of DMSO was added to the mixture and kept stirring for 4 h at 70 °C. Afterwards, the crosslinked product was precipitated in 30 mL isopropanol, filtered and washed. Thereafter, it was neutralized and freeze dried before measuring water absorption and other characterization.

### 3.2. Characterization

#### 3.2.1. ATR-FTIR Spectroscopy

Attenuated total reflection Fourier transform Infra-red (ATR-FTIR) spectra of raw materials (BF and Ok) and the mechanochemically synthesized SAPs were recorded using Thermo Fisher Scientific Nicolet iS10 (Thermo Fisher Scientific, Inc., Tokyo, Japan) spectrophotometer with an average scans over 64 at a resolution of 4 cm^−1^ in the range from 400 to 4000 cm^−1^.

#### 3.2.2. X-Ray Diffraction Analysis

The X-ray diffraction analysis of raw biomass (BF and OK) as well as mechanochemically synthesized SAPs was performed using D2 Phaser 2nd Gen diffractometer (Bruker, Karlsruhe, Germany) having Cu-Kα radiation (λ = 0.154 nm) in the range 2θ = 5–60°.

#### 3.2.3. Surface Morphology

Scanning electron microscopy (SEM) images of raw biomass and SAPs were examined at operating voltage of 10 kV on a JEOL JSM-6510LV scanning electron microscope ((JSM-6510LV, JEOL Ltd., Akishima, Tokyo, Japan). Before recording micrographs, the samples were mounted on aluminium stubs with conductive carbon tape and sputter-coated with the thin layer of platinum using Magnetron sputtering equipment (MSP-20UM, Vacuum Device Inc., Ibaraki, Japan) under high vacuum to ensure electrical conductivity. The images were obtained under high vacuum at magnifications ×500.

#### 3.2.4. Measurement of Functionalization of BF/Okara

For measurement of carboxyl functionalities introduced into the polysaccharide’s backbone structure of BF and Ok during succinoylation, acid–base titration method was adopted from the literature. In a 100 mL glass bottle equipped with magnetic stirrer, 0.5 g succinoylated polysaccharide materials were dispersed in 10 mL distilled water at 75 °C for 30 min. Afterwards, it was titrated with 0.1 M NaOH standard solution using phenolphthalein as an indicator. The number of -COOH groups were calculated by the formula given in Equation (2):

(2) $$COOH\;(\mathrm{mmol}/g)=\frac{C_{NaOH}\times V_{NaOH}}{m}$$

where $C_{NaOH}$ is the concentration of NaOH solution (mol/L), $V_{NaOH}$ is the volume of NaOH used for titration (mL), and *m* is the mass (g) of succinoylated material.

### 3.3. Measurement of Free Water Uptake

The free water uptake of the synthesized SAPs was studied using the Japanese industrial standard tea bag method [58]. Nylon fabric having a pore size of 57 µm (255 mesh) was used to prepare tea bags of 10 × 20 cm^2^ size by heat sealing. Then, carefully weighed 0.2 g SAP samples were placed in the tea bags and put in 1 L of water. After 3 h the tea bags were removed from water and hung in the air with the help of clips to drain excess water for 10 min. The weight gain by the SAPs by up taking the water was measured and swelling ratio *Q* was calculated by Equation (3) as follows:

(3) $$Q=\frac{W_{SAP}-W_{bag}-W_{0}}{W_{0}}$$

where *Q* is SAP swelling ratio (g/g); *W*_SAP_ is weight of swollen SAP (g) in a tea bag; *W_bag_* is average weight of three wet tea bags (g); and *W*_0_ is weight of dried SAP (g).

### 3.4. Growth of Maize Plants

The SAP (Entry 9) prepared using twin-screw extruder was used to evaluate the maize (*Zea mays*) plant growth. It was selected because it was produced through continuous twin-screw extrusion without a catalyst, representing a simpler and more scalable preparation approach. Although it did not show the highest water absorption, its performance was sufficient for evaluating its potential as a directly produced soil amendment. Maize seeds were germinated in the dish using moist tissue paper (Figure S2) for 3 days at room temperature prior to transplantation. The growth experiment was conducted using 220 mL polyester transparent cups having holes at the bottom and filled with 70 g of vegetable garden soil (DCM Brand, Japan). The SAPs (purified from unreacted SA and neutralized as well as unpurified and non-neutralized, henceforth called treated and untreated, respectively) were incorporated into the soil at concentration of 0.1 wt.%, 0.2 wt.% and 0.4 wt.%. Control samples without SAP were also prepared for comparison. Two germinated seeds were sown per cup at a depth of approximately 2–3 cm. Five replicate cups were prepared for each concentration of SAP. All cups were maintained under controlled laboratory growth chambers (temperature: 25.5 °C; relative humidity: ~54%; 16 h light/8 h dark cycle). To assess the effect of SAPs on maize plants grown in SAP-added soil, water (50 mL) was added once to the cups after sowing the seeds and excess water was drained off through bottom holes. After 10 days of this preliminary and exploratory growth experiment, plants were carefully uprooted from the soil and roots were cleaned of dirt. The height of the plants and length of the roots was measured to assess the effect of the SAP on plant growth. Statistical analysis was performed for each SAP concentration on maize roots and shoots to find the significantly different groups. The additional percentage effect on maize roots and shoots for each SAP concentration relative to control was calculated by using the average shoots and roots length by using the formula:

(4) $$Percentage\;Effect\;(\%)=\left(\frac{L_{sample}-L_{control}}{L_{sample}}\right)\times 100$$

where *L_sample_* represents the mean length of roots and shoots of the SAP amended maize plants group and *L_control_* represents the mean length of the control maize plants group.

### 3.5. Statistical Analysis

All experiments were performed in replicates, and the results are presented as mean ± standard deviation. For the maize growth experiment, five independent cups were prepared for each treatment, with two plants cultivated in each cup. Measurements from the two plants within each cup were averaged, and the cup mean was considered one independent replicate (*n* = 5). Statistical analyses were performed using OriginPro 2026 (OriginLab Corporation, Northampton, MA, USA). Differences in maize plants growth of shoots and roots with concentrations of untreated SAP and treated SAP were evaluated by one-way analysis of variance (ANOVA) followed by Tukey’s honestly significant difference (HSD) multiple-comparison test. Statistical significance was considered at *p* < 0.05. The resulting statistical significance groupings were subsequently mapped directly onto the calculated percentage effect graph, while the cumulative variance of both the treated and control samples on the percentage-effect graph, error bars were determined via relative error propagation.

## 4. Conclusions

In conclusion, this study presented a mechanochemical approach to functionalize BF and okara polysaccharides with -COOH functionalities to prepare SAPs. Two catalysts, K_2_CO_3_ and Zn(OAc)_2_, were tested for effective increase in -COOH functionalities during the reactive extrusion process. It was observed that K_2_CO_3_ proved to be an effective catalyst in increasing the functionalities while Zn(OAc)_2_ showed slight increase in functionalities. Among the basic solutions used for conversion of carboxylic functionalities to carboxylate salts, aq. KOH neutralized SAPs showed better water absorption. The use of crosslinkers, GMA and MBA, reduced the water absorption of SAPs probably due to the crosslinking mechanism. Among GMA and MBA, the latter showed better performance of water absorption as compared to GMA. The SAP produced on a large scale using a continuous a co-rotating twin-screw extruder was used to grow maize plants and the results indicated that untreated and treated SAPs, particularly at 0.4 wt.% showed better growth than the control. So, the SAPs can be produced on a large scale with potential application in sustainable agriculture.

## Figures and Tables

**Table 1:** Feed composition for synthesis of SAPs, amounts of catalysts, and the weight percent gain (WPG) after mechanochemical functionalization reaction.

SAPs	Raw Materials (g)				K_2_CO_3_ Catalysed	Zn(OAc)_2_ Catalysed
	BF	Okara	SA	Catalyst ^1^	WPG(%)	WPG(%)
Entry 1	3.2	-	10.3	-	95.0	95.0
Entry 2	-	3.2	10.3	-	79.1	79.1
Entry 3	1.6	1.6	10.3	-	43.8	43.8
Entry 4	1.6	1.6	10.3	0.16	77.2	39.1
Entry 5	1.6	1.6	10.3	0.32	88.8	47.8
Entry 6	1.6	1.6	10.3	0.48	98.1	59.7
Entry 7	1.6	1.6	10.3	0.64	111.3	50.9
Entry 8	1.6	1.6	10.3	0.75	137.8	65.0
Entry 9	1.6	1.6	10.3	-	17.5	17.5

^1^ The amount of catalysts: K_2_CO_3_ and Zn(OAc)_2_ were used in separate reactions.

**Table 2:** Water absorption (g/g) of SAPs after neutralization with KOH, aq. NH_3_ and NaOH.

SAPs	Water Absorption (g/g) ^3^			
	K_2_CO_3_ Catalysed		Zn(OAc)_2_ Catalysed	
	KOH	aq. NH_3_	KOH	NaOH
Entry 1	13.9 ± 2.4 ^a^	10.2 ± 2.2 ^a^	13.9 ± 2.4 ^a^	12.1 ± 1.6 ^a^
Entry 2	19.1 ± 4.2 ^a^	13.7 ± 3.9 ^a^	19.1 ± 4.2 ^a^	15.2 ± 2.6 ^a^
Entry 3	15.6 ± 2.1 ^a^	10.7 ± 1.7 ^a^	15.6 ± 2.1 ^a^	13.8 ± 1.3 ^a^
Entry 4	34.9 ± 1.2 ^b^	14.4 ± 2.3 ^a^	17.1 ± 1.7 ^a^	14.0 ± 2.4 ^a^
Entry 5	35.9 ± 2.4 ^b^	17.6 ± 4.4 ^ab^	17.5 ± 2.1 ^a^	16.7 ± 2.3 ^a^
Entry 6	33.9 ± 1.4 ^b^	24.1 ± 2.9 ^b^	18.1 ± 2.8 ^a^	17.0 ± 1.3 ^a^
Entry 7	36.5 ± 2.2 ^b^	35.8 ± 2.3 ^c^	19.9 ± 1.2 ^a^	17.2 ± 1.1 ^a^
Entry 8	34.9 ± 1.5 ^b^	17.7 ± 2.5 ^ab^	16.3 ± 2.7 ^a^	17.0 ± 2.2 ^a^
Entry 9	14.5 ± 1.3 ^a^	8.2 ± 0.7 ^a^	14.5 ± 1.3 ^a^	13.8 ± 1.2 ^a^

^3^ The values of water absorption are presented as mean ± standard deviation of triplicate (*n* = 3). Different superscript letters within the same column indicate statistically significant differences at *p* < 0.05 according to Tukey’s HSD test. The same superscript letter within the column indicates no statistically significant difference at *p* < 0.05 according to Tukey’s HSD test.

**Table 3:** Comparative overview of water uptake capacities for various biomass-derived and hybrid superabsorbent materials.

Materials Composition	Maximum Water Uptake (g/g)	Classification/Context	References
BF/Okara succinate	~36.5	Superabsorbent	This work
Commercial synthetic SAPs (in saline)	<100	Superabsorbent	[64,65]
Starch-based sulphate	34–176	Superabsorbent	[65]
polysaccharide–polyvinyl alcohol–montmorillonite clay nanocomposites (in saline)	40	Superabsorbent	[66]
Starch-carboxymethyl cellulose based	87	Superabsorbent	[67]
Agricultural residue	79	superabsorbent	[68]
Okara itaconate	~35	Superabsorbent	[57]

## Data Availability

The original contributions presented in this study are included in the article/Supplementary Material. Further inquiries can be directed to the corresponding authors.

## Supplementary Materials

The following supporting information can be downloaded at: https://www.mdpi.com/article/10.3390/ijms27188097/s1.

## Abbreviations

The following abbreviations are used in this manuscript:

ATR-FTIR	Attenuated total reflectance-Fourier transform Infra-red
BF	Banana pseudo-stem fibres
GMA	Glycidyl methacrylate
K_2_CO_3_	Potassium carbonate
MBA	*N*,*N*′-methylene *bis*acrylamide
SA	Succinic anhydride
SAPs	Superabsorbent polymers
SEM	Scanning electron microscopy
XRD	X-ray diffraction
Zn(OAc)_2_	Zinc acetate
