# Supplementary material for: Mechanochemical Functionalization of Polysaccharides via Catalytic Esterification for the Preparation of Superabsorbent Materials for Plant Growth Applications
Source: Int J Mol Sci. 2026 Sep 11;27(18):8097. doi: 10.3390/ijms27188097 (PMC13606771; doi:10.3390/ijms27188097)
Supplement: Supplementary file 1 [file ijms-27-08097-s001.zip › ijms-4389076-supplementary.pdf]

Supplementary Information

Mechanochemical Functionalization of Polysaccharides via Catalytic Esterification for the Preparation of Superabsorbent Materials for Plant Growth Applications

Abdul Hafeez [1,*], Hao Wang [1], Romain Milotskyi [2], Gyanendra Sharma [2], Naoki Wada [2] and Kenji Takahashi [2,*]

[1] Division of Biological Science and Technology, Graduate School of Natural Science and Technology, Kanazawa University, Kakuma-Machi, Kanazawa 920-1192, Ishikawa, Japan;

[2] Faculty of Biological Science and Technology, Institute of Science and Engineering, Kanazawa University, Kakuma-Machi, Kanazawa 920-1192, Ishikawa, Japan;

* Correspondence: hafeez163@stu.kanazawa-u.ac.jp (A.H.); ktkenji@staff.kanazawa-u.ac.jp (K.T.); Tel.: +81-90-1395-4507 (A.H.); +81-76-234-4828 (K.T.)

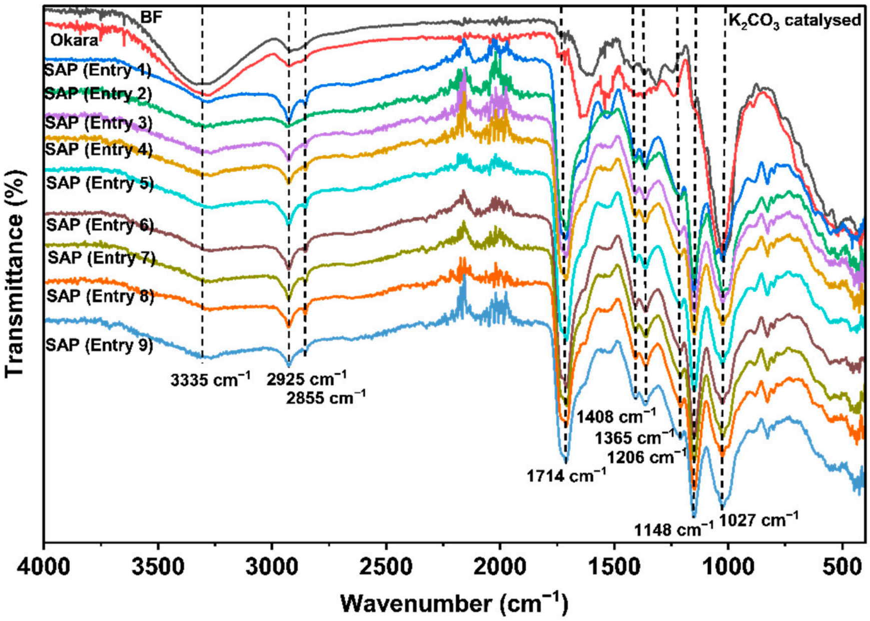

Figure S1. ATR mode FTIR of succinoylated SAPs before neutralization showing single broad peak arising due to merging of ester and carboxylic acid peaks.

Table S1. Carboxyl content (mmol/g) of SAPs prepared by K_2CO_3 and $Zn(OAc)_2$ catalysis and statistical differences using One-Way ANOVA followed by Tukey's HSD test.

SAPs	Catalyst (g)	COOH content (mmol/g) in K_2CO_3 catalysed SAPs	COOH content (mmol/g) in $Zn(OAc)_2$ catalysed SAPs
Entry 1	-	3.30 ± 0.01^{ab}	3.30 ± 0.01^{xy}
Entry 2	-	3.65 ± 0.07^{bcd}	3.65 ± 0.07^{y}
Entry 3	-	2.93 ± 0.11^{a}	2.93 ± 0.11^{x}
Entry 4	0.16	3.94 ± 0.33^{bcde}	3.35 ± 0.58^{xy}
Entry 5	0.32	4.07 ± 0.41^{cde}	3.38 ± 0.35^{xy}
Entry 6	0.48	4.21 ± 0.43^{def}	3.64 ± 0.09^{y}
Entry 7	0.64	4.63 ± 0.22^{ef}	3.53 ± 0.25^{xy}
Entry 8	0.75	4.80 ± 0.40^{f}	3.58 ± 0.31^{xy}
Entry 9	-	3.46 ± 0.33^{abc}	3.46 ± 0.33^{xy}

Data are presented as mean ± standard deviation ($n = 4$). Different superscript letters within the same column indicate statistically significant differences at $p < 0.05$ according to Tukey's HSD test.

Table S2. Carboxyl content (mmol/g) of SAPs prepared by K_2CO_3 and $Zn(OAc)_2$ catalysis and statistical differences using Two-Way ANOVA followed by Tukey's HSD test.

SAPs	Catalyst (g)	COOH content (mmol/g) in K_2CO_3 catalysed SAPs	COOH content (mmol/g) in $Zn(OAc)_2$ catalysed SAPs
Entry 1	-	3.30 ± 0.01^{ab}	3.30 ± 0.01^{ab}
Entry 2	-	3.65 ± 0.07^{abcd}	3.65 ± 0.07^{abcd}
Entry 3	-	2.93 ± 0.11^{a}	2.93 ± 0.11^{a}
Entry 4	0.16	3.94 ± 0.33^{bcde}	3.35 ± 0.58^{abc}
Entry 5	0.32	4.07 ± 0.41^{cdef}	3.38 ± 0.35^{abc}
Entry 6	0.48	4.21 ± 0.43^{def}	3.64 ± 0.09^{abcd}
Entry 7	0.64	4.63 ± 0.22^{ef}	3.53 ± 0.25^{abcd}
Entry 8	0.75	4.80 ± 0.40^{f}	3.58 ± 0.31^{abcd}
Entry 9	-	3.46 ± 0.33^{abcd}	3.46 ± 0.33^{abcd}

Data are presented as mean ± standard deviation ($n = 4$). Different superscript letters across the entire table indicate statistically significant differences between any two formulations at $p < 0.05$ according to a Two-Way ANOVA followed by Tukey's HSD test.

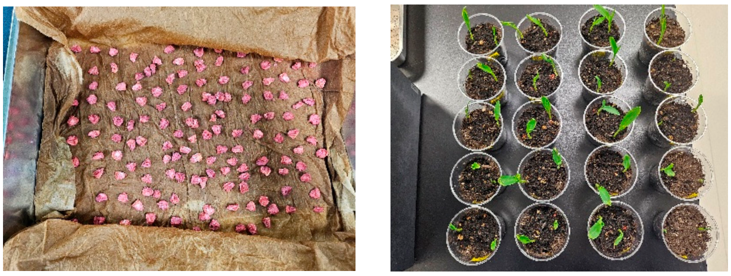

(**a**) (**b**)

Figure S2. (a) Maize seeds placed in soaked tissue paper for germination, (b) growth of maize plants on 4th Day in untreated SAP, rows from left to right: 0.1 wt% SAP, 0.2 wt% SAP, 0.4 wt% SAP and control.

Table S3. Water absorption (g/g) of SAPs synthesized using K_2CO_3 catalyst, neutralized with NaOH and crosslinked with GMA and MBA crosslinker.

SAPs	Water absorption (g/g) of GMA crosslinked SAPs	Water absorption (g/g) of MBA crosslinked SAPs
Entry 1	16.9±1.7	19.2±1.7*
Entry 2	16.9±1.4	17.5±1.2*
Entry 3	14.8±1.3	20.6±1.6*
Entry 4	17.1±1.5	24.9±1.3*
Entry 5	19.6±1.4	29.6±1.8*
Entry 6	19.9±1.6	30.4±1.5*
Entry 7	23.0±1.9	31.1±1.8*
Entry 8	18.5±1.2	29.4±1.4*
Entry 9	10.5±1.1	21.4±1.2*

Data are presented as mean ± standard deviation ($n = 3$). Two-way ANOVA followed by Tukey's HSD post-hoc test, indicated that overall, MBA crosslinked SAPs exhibited significantly higher water absorption than GMA crosslinked SAPs ($p < 0.0001$).
